# CD133 Is Not Suitable Marker for Isolating Melanoma Stem Cells from D10 Cell Line

**DOI:** 10.22074/cellj.2016.3983

**Published:** 2016-04-04

**Authors:** Motahareh Rajabi Fomeshi, Marzieh Ebrahimi, Seyed Javad Mowla, Javad Firouzi, Pardis Khosravani

**Affiliations:** 1 Department of Developmental Biology, University of Science and Culture, ACECR, Tehran, Iran; 2 Department of Stem Cells and Developmental Biology, Cell Science Research Center, Royan Institute for Stem Cell Biology and Technology, ACECR, Tehran, Iran; 3 Department of Molecular Genetics, Faculty of Biological Sciences, Tarbiat Modares University, Tehran, Iran

**Keywords:** Melanoma, Cancer Stem Cell, CD133, Spheroid

## Abstract

**Objective:**

Cutaneous melanoma is the most hazardous malignancy of skin cancer with a high mortality rate. It has been reported that cancer stem cells (CSCs) are responsible for malignancy in most of cancers including melanoma. The aim of this study is to compare two common methods for melanoma stem cell enriching; isolating based on the CD133 cell surface marker and spheroid cell culture.

**Materials and Methods:**

In this experimental study, melanoma stem cells were enriched by fluorescence activated cell sorting (FACS) based on the CD133 protein expression and spheroid culture of D10 melanoma cell line,. To determine stemness features, the mRNA expression analysis of *ABCG2, c-MYC, NESTIN, OCT4-A* and *-B* genes as well as colony and spheroid formation assays were utilized in unsorted CD133^+^, CD133^-^ and spheroid cells. Significant differences of the two experimental groups were compared using student’s t tests and a two-tailed value of P<0.05 was statistically considered as a significant threshold.

**Results:**

Our results demonstrated that spheroid cells had more colony and spheroid forming ability, rather than CD133^+^ cells and the other groups. Moreover, melanospheres expressed higher mRNA expression level of *ABCG2, c-MYC, NESTIN* and *OCT4-A* com- pared to other groups (P<0.05).

**Conclusion:**

Although CD133^+^ derived melanoma cells represented stemness fea- tures, our findings demonstrated that spheroid culture could be more effective meth- od to enrich melanoma stem cells.

## Introduction

Cutaneous melanoma is derived from transformation of the pigment-producing melanocytes (1), leading to highest mortality rate of skin cancers. Several studies suggest the presence and involvement of cancer stem cells (CSCs) in initiation, propagation, invasion, chemoresistance and therapeutic failure of this malignancy (2,3). Therefore, identifying melanoma SCs as well as related molecular activities, and regulatory pathways might serve to develop a new therapeutic strategy to treat progression of this lethal form of disorder. Isolation of CSCs based on cell surface markers is of the most feasibly approaches to yield high amount of particular cells. It has been revealed that CD133 protein, as a critical CSC-associated marker generally expressed in some specific SCs including hematopoitic SCs, can also play crucial role in various cancer types such as cutaneous melanoma (4). Further investigations demonstrated that injection of CD133^+^ melanoma cells could lead to develop a detectable tumor in mice, whereas no tumor development was recognized by injection of CD133^–^ melanoma cells (5). Moreover, down-regulation of CD133 resulted in lower rate of cell growth and reduced level of cell motility (6). Spheroid formation is another method for CSCs enrichment, whereby the cells are cultured in serum-free medium and low-attachment plates (7,8). Usually, epidermal growth factor (EGF) and basic fibroblast growth factor (bFGF) are added to support CSCs growth. Thus far, it is not clear which of the indicated methods could be more practical and beneficial to isolate the melanoma SCs. Here, we compared two indicated methods to determine the feasibility of melanoma SCs isolation. 

## Materials and Methods

### Cell lines and culture conditions

In this experimental study, the human melanoma cell line D10 was generously provided by Prof. Giulio Spagoli (University Hospital of Basel, Switzerland). In adhesive condition, 15×10^4^cells were cultured per T25 cell culture flask in a complete Roswell Park Memorial Institute (RPMI)-1640 base medium, supplemented with 10% fetal bovine serum (FBS), 1% non-essential amino acids (NEAA), 2mM L-glutamine, 1% penicillin/streptomycin (all were purchased from Gibco, Germany). The cells were incubated at 37˚C temperature and 5% CO_2_atmosphere conditions. The media was refreshed every 2 days and the cells were passaged every 6 days. In spheroid condition, 30×10^3^cells/ml was grown in T25 culture flask, coated with 12-mg/ml poly 2-hydroxyethyl methacrylate (polyHEMA, Sigma, Germany). Serum free (SF) RPMI containing 1% NEAA, 2 mM L-glutamine, 1% penicillin/streptomycin, 1x B-27 supplement (Gibco, Germany), 20 µg/ml of each EGF and bFGF (Royan, Iran) were used as culture medium of spheroid culture. Cells were cultured for 6 days and the fresh B27, bFGF and EGF were added to culture medium every 2 days. 

## Flow cytometry analyzing and fluorescenceactivated cell sorting

For flow cytometry analyzing, almost 1.5×10^5^adherent cells were harvested by 0.05% trypsinEthylenediaminetetraacetic acid (EDTA, Gibco, Germany) and incubated with CD133-phycoerythrin (PE, Miltenyi Biotec., Germany), followed by antibody labeling and IgG antibody isotypematching for 45 minutes at 4˚C. The cells were then washed and re-suspended in Dulbecco’s phosphate-buffered saline (DPBS) buffer (Gibco, Germany). Positive cell filed was determined as percentage, using fluorescence-activated cell sorting (FACS) calibur machine (Becton Dickinson, Belgium). The results were analyzed by flowing software version 2.5.0. 

For cell sorting, adherent cells were harvested by 0.05% trypsin-EDTA and washed in DPBS containing 2% FBS. the cells were subsequently resuspend in 500 μl sorting buffer containing DPBS supplemented with 2 mM EDTA (Sigma, Germany), 25 mM N-2-hydroxyethylpiperazine-N-2-ethane sulfonic acid (HEPES, Sigma, Germany) and 1% FBS, followed by staining with 3 µl/10^6^cells of mouse-anti-CD133 (clone 293C3)-PE monoclonal antibody (Miltenyi Biotec, Germany). PE-labeled mouse IgG2b (Miltenyi Biotec. Germany) was applied as background control for nonspecific binding. The cells were incubated for 45 minutes on ice in dark condition, then washed with PBS containing 2% FBS. They were next re-suspended in sorting buffer at density 2×10^6^cells/1 ml and then sorted with FACSAriaII (Becton Dickinson, USA). Ultimately, 5000-sorted cells were reanalyzed for each group. 

## Colony formation assay

The colony formation assay was performed on the D10, spheroids, CD133^+^ , and CD133^-^ cells. For each group, 200 survived cells/well were incubated in 6-well plates, containing 2.5 ml complete medium per well, followed by incubation for 10 days. The complete medium contains RPMI-1640 base medium, supplemented with 10% FBS, 1% NEAA, 2 mM L-glutamine, 1% penicillin/streptomycin. The colonies with >40 cells were subsequently counted under an invert microscope at ×40 magnification. 

## Sphere formation assay

The sphere formation assay was also performed on the D10, spheroids, CD133^+^ , and CD133^-^ cells. Cells were plated at a density of 5,000 cells/well in ultra-low attachment 6-well plates and grown in 2 ml SF RPMI containing 2 mM L-glutamine, 1% penicillin/streptomycin, 1% NEAA. 20 ng/ml EGF and bFGF were added every 48 hours to the culture medium. The experiments were performed in triplicate and three different biological replicates. Plates were maintained in 5% CO_2_ incubator at 37˚C for 10 days. The numbers of spheres with >20 cells were counted under a microscope at ×40 magnification. 

## Real-time quantitative reverse transcriptionpolymerase chain reaction analysis

Total RNA was extracted from different groups using TRIzol reagent (Sigma, Germany). Next, cDNA was synthesized using random hexamers by cDNA Synthesis Kit (TaKaRa, Germany) according to the manufacturer’s instructions. cDNA products were diluted eight times and then subjected to real-time quantitative RT-PCR (qRT-PCR) via Corbet Life Science real-time PCR System RotorGene 6000 (Series Software 1.7, UK). Reactions were performed according to the manufacturer’s instructions by using SYBR Green PCR Master Mix (Biosystems, UK) and human-specific primers included: *CD133, OCT4-A, OCT4-B, c-MYC, NESTIN* and *ABCG2*. *GAPDH* was used as the internal control for normalization of all reactions. 

The applied forward (F) and reverse (R) primers were as follow:

CD133 -F: 5´-GCATCCATCAAGTGAAACGT-3´
CD133 -R: 5´-GGTTTGGCGTTGTACTCTG-3´
OCT4-A -F: 5´-CTGGGTTGATCCTCGGACCT-3´
OCT4-A -R: 5´-CACAGAACTCATACGGCGGG-3´
OCT4-B -F: 5´-GTTCTTCATTCACTAAGGAAGG-3´
OCT4-B -R: 5´-CAAGAGCATCATTGAACTTCAC-3´
c-MYC -F: 5´-ACACATCAGCACAACTACG-3´
c-MYC -R: 5´-CGCCTCTTGACATTCTCC-3´
NESTIN -F: 5´-TCCAGGAACGGAAAATCAAG-3´
NESTIN -R: 5´-GCCTCCTCATCCCCTACTTC-3´
ABCG2 -F: 5´-CCACTCCCACTGAGATTGAG-3´
ABCG2 -R: 5´-CAAACAAACTCTAAAGCAGC-3´
GAPDH -F: 5´-CTCATTTCCTGGTATGACAAC-3´
GAPDH -R: 5´-CTTCCTCTTGTGCTCTTGCT-3´

All samples were run in duplicate and repeated three times. PCR condition was set as 95˚C for 10 minutes, 40 cycles of denaturation at 95˚C for 10 seconds, annealing at 60˚C for 20 seconds, and elongation fluorescence monitoring at 72˚C for 20 seconds. A final melting curve analysis was performed from 65˚C to 95˚C and data analyzed by 2^-ΔΔCt^ method. Expression of these genes was analyzed in the D10, CD133^+^, CD133^-^ and spheroids cells.

## Statistical analysis

Most assays were performed in triplicate and repeated three times. The differences between the two experimental groups were determined using Student’s t tests. A two-tailed value of P≤0.05 was considered statistically significant. 

## Results

### Morphological future of the D10 cells in adherent and spheroid culture conditions

**Fig.1:**
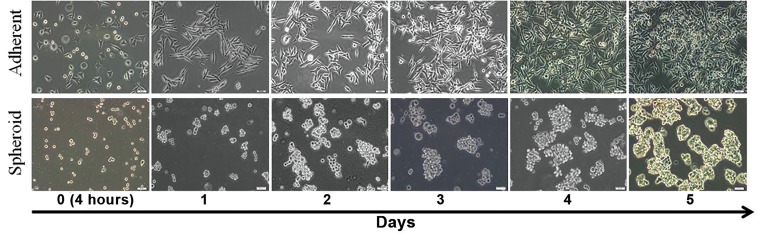
D10 cells morphology in adherent (up) and spheroid (down) culture at days 0, 1, 2, 3, 4 and 5 (scale bar: 50 μm).

Morphologically, the D10 cells in adherent culture condition had elongated or formed a spindle shape, whereas in spheroid culture, they had aggregated loosely with rounded or "amoeboid-like" shape (Fig.1). 

### D10-melanoma stem cells clonogenicity and tumorigenicity *in vitro*

**Fig.2:**
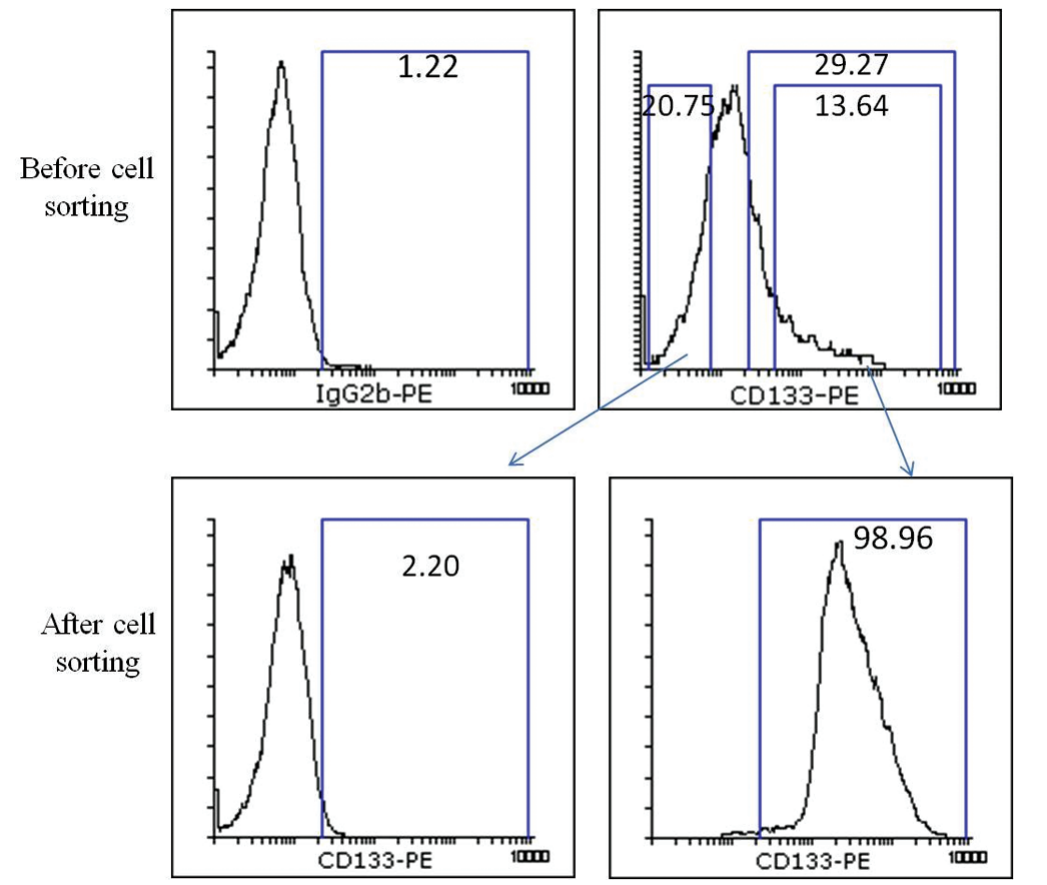
Separation of CD133^+^ and CD133^-^ cells from the D10 cell line by FACSAria II. Percentages indicate the frequency of cells that stained more strongly in comparison with the isotype control (left). Re-analysis of sorted cells is shown at the bottom row; i.e. after cell sorting.

Spheroid formation and cell sorting based on CD133 expression were applied to enrich melanoma SCs. In spheroid culture condition, CD133 protein was expressed in approximately 29.27 ± 2.83% of the D10 cells. As shown in figure 2, we selected 20.75% of the CD133^-^ and 13.64% of the CD133^+^ cells for sorting and the populations with greater than 95% purity were subsequently selected for future assays (Fig.2).

**Fig.3:**
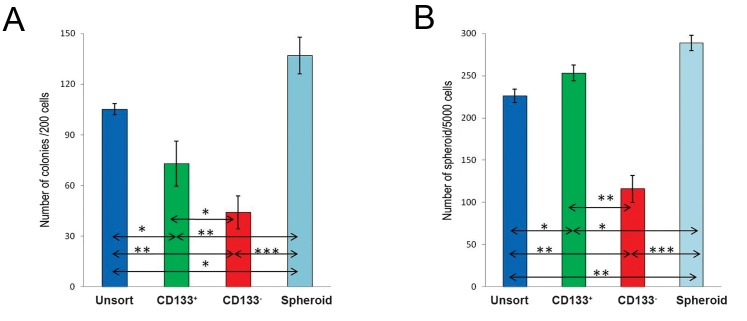
A. Number of Colony formation with 200 initial cells seeding in 6 well plates and B. Number of spheroid formation in each group with 2500 viable cells/ml (5000cells/well), after 10 days.*; P≤0.05, **; P≤0.005 and ***; P≤0.0005.

We used colony and spheroid formation assays in enriched cells to determine tumorigenicity and self-renewal capacities *in vitro*. Results demonstrated more colonies and spheroids, in terms of the quantity, in CD133^+^ compared to CD133^-^ cells. However, spheroid cells had the greatest number of colonies and spheres (P≤0.05, Fig.3A, B).

### Differentially expression of stemness related markers in the D10-melanoma stem cells 

**Fig.4:**
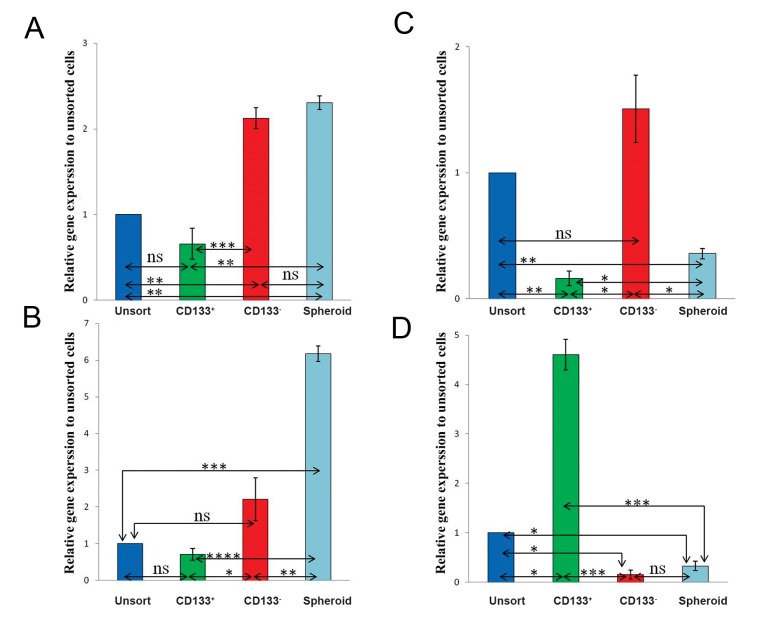
Real time quantitative reverse transcriptase-polymerase chain reaction analysis of A. *c-MYC*, B. *NESTIN*, C. *ABCG2* and D. *CD133* expression in the unsorted/adherent D10, CD133^+^, and CD133^-^ fractions and spheroid cells. All experiments were done in duplicate and repeated three times and normalized to GAPDH; values are expressed as means ± SD. *; P≤0.05, **; P≤0.005, ***; P≤0.001, ****; P= 7.36×10^6^ and ns; Non-significant.

qRT-PCR analyses, among the tested genes indicated that *c-MYC* and *ABCG2* mRNA expression levels were increased in melanospheroieds, with lower extent to CD133^-^ and CD133^+^ cells (P≤0.05, Fig.4A, B). In contrast to the other groups, the mRNA expression of *NESTIN* gene was significantly up-regulated in CD133^-^ cells (Fig.4C). CD133 was the only transcriptionally over-expressed gene in CD133^+^ cells (P≤0.05, Fig.4D). Comparing two enriched populations reveled that expression of *ABCG2, NESTIN* and *c-MYC* was significantly up-regulated in melanoma-sphere cells rather than CD133^+^ . 

### Differentially expression of OCT4 variants in CD133^+^ and spheroid cell populations

**Fig.5:**
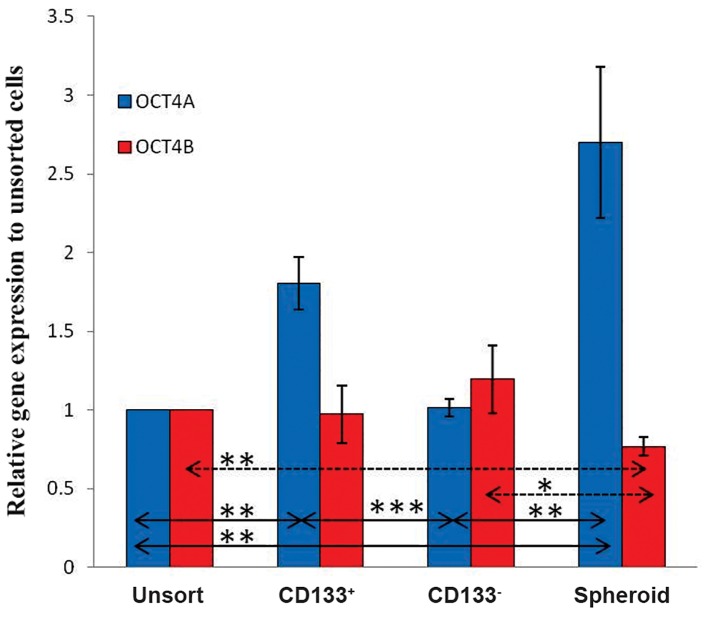
Real time quantitative reverse transcriptase-polymerase chain reaction analysis of *OCT4-A* and *OCT4-B* expression in the unsorted/adherent D10, CD133^+^, and CD133^-^ fractions and spheroid cells. All experiments were done in duplicate and repeated three times and normalized to GAPDH; values are expressed as means ± SD. *; P≤0.05, **; P≤0.03 and ***; P≤0.01.

In this study, we assessed the mRNA expression of two OCT4 gene variants, including *OCT4-A* and *OCT4-B*. Findings showed in Figure 5 that spheroid cells expressed higher level of *OCT4A* mRNA expression compared to CD133^+^ cells (P≤0.05). *OCT4-B* expression was down-regulated in spheroids compared to unsorted and CD133^-^ cells, however, no significant difference was observed by comparing mRNA expression level of these variants in CD133^+^ cells with the other groups (Fig.5). 

## Discussion

CSCs involve in tumor initiation and progression as well as chemoresistance and therapeutic failure in human malignant melanoma (2). Hitherto, several methods have been used for identification and characterization of melanoma stem cells (5,9). Here, we compared two common methods which are used for CSCs enrichment; one based on the expression of CD133 protein, and the other sphere formations. To confirm the stemness propensity, colony and sphere formation capacities as well as mRNA expression of several stem cell markers were assessed in both enrichment methods. 

The results of colony and sphere formation assays, reflecting self-renewal and tumor initiation capacities, demonstrated that melanoma spheres were more clonogenic with higher spheroid formation ability than other groups. These data were compatible with other studies indicating self-renewal capacity of melanoma cells affected by microenvironment (10,11). 

*c-MYC, ABCG2, NESTIN* and *OCT4* have important role in cancer progression (12,15). Among these genes only *c-MYC, ABCG2* and *OCT4-A* were up-regulated in melanospheres. Studies introduced c-MYC oncoprotein as a prognostic marker in melanoma (10) which induces melanoma cell growth (11,12). Members of the ATP-binding cassette (ABC) transporter protein family have also been reported to be expressed in melanoma stem cells. These transmembrane proteins hydrolyze ATP to perform some functions including transportation of various substrates (15). Out of four, two ABC transporter protein expressions, ABCG2 and ABCB5, have been identified in potential melanoma stem cells. Moreover, ABCG2 provides a mechanism of resistance to a wide variety of drugs, including the tyrosine kinase inhibitors imatinib and gefitinib, antibiotics, and HMG-CoA inhibitors (16,17). 

*OCT4*, as one of embryonic stem/germ cell markers, is responsible for self-renewal, cell maintenance and pluripotency (18,19). Two major isoforms have been determined for this protein, including *OCT4-A* and *OCT4-B*. *OCT4-A* is only presented isoform in the pluripotent state, whereas no biological function has been detected for *OCT4-B* (13). Elevated levels of *OCT4* in tumors have been associated with metastases and shorter patient survival rates (14). 

Melanoma SCs might potentially form spheroid shapes. In contrast to the others, some studies demonstrated that NESTIN, as neural stem cell marker, is not up-regulated in this type of spheroid cells as potent melanoma stem cells. In addition, expression of NESTIN has been indicated as a poor prognostic factor of melanoma cancer (15). Moreover, in this enriched group (melanospheres), CD133 and *OCT4-B* were down-regulated. Monzani et al. (5) found that melanoma sphere-associated cells did not express CD133 protein. In contrast Perego et al. (20) observed that melanospeheres highly expressed CD133 and ABCG2 proteins. 

Considering the previous controversial reports, findings of current study suggest that sphere culture condition, containing serum-free medium, EGF, bFGF, and B-27 at the low attachment plate, could be more reasonable method to obtain enriched melanoma SCs from CD133^+^ sorted cells. Although several studies have demonstrated that CD133 protein expression could be increased in a wide range of human cell malignancies, including metastatic lesions or tumor recurrent for cancer patients, it could also be over-expressed in malignancy (4,9) or cancer stem cell derived from melanoma samples (5). Most of the previous studies, emphasizing on CD133 protein as a marker for melanoma stem cells, had been performed on the human patient tissue biopsies (5,7,8). In contrast, this study employs melanoma cell lines to sort and enrich the CSCs. Our findings demonstrated different levels of CD133 expressions *in vitro*, suggesting that this protein could likely not be a suitable marker to apply in isolation of CSCs. This expression level diversity could be observed due to the changes in different conditions like incubation time, passaging, pH and the other factors which might not be appropriately controlled *in vitro*. 

## Conclusion

This study highlights that spheroid culture condition can be a more effective approach to enrich cancer stem cells, rather than CD133-based cell sorting. This melanospheres have also more stemness feature than CD133^+^ cells. 
